# Supplementary material for: Socioeconomic deprivation and illness trajectory in the Scottish population after COVID-19 hospitalization
Source: Commun Med (Lond). 2024 Feb 28;4:32. doi: 10.1038/s43856-024-00455-5 (PMC10901805; doi:10.1038/s43856-024-00455-5)
Supplement: Supplementary file 2 — Description of Additional Supplementary Files [file 43856_2024_455_MOESM2_ESM.pdf]

Description of Additional Supplementary Files

File Name: Supplementary Data 1
Description: Clinical characteristics of the study population, by deprivation status. Quintile 1 = most deprived, Quintile 5 = least deprived

File Name: Supplementary Data 2
Description: Multisystem phenotypying by deprivation: serial electrocardiography, biomarkers of inflammation, metabolism, renal function,
and haemostasis, and heart, lung, and kidney imaging at 28-60 days post-discharge

File Name: Supplementary Data 3
Description: Clinical characteristics of the study population, by attendance at Visit 2.

File Name: Supplementary Data 4
Description: Health status, illness perception, anxiety and depression, and physical function by deprivation status.

File Name: Supplementary Data 5
Description: Linear mixed effects regression models for patient reported outcomes in relation to SIMD

File Name: Supplementary Data 6
Description: Linear mixed effects regression model data for patient reported outcomes in relation to SIMD

File Name: Supplementary Data 7
Description: Clinical outcomes by deprivation status.
