# Supplementary material for: Socioeconomic deprivation and illness trajectory in the Scottish population after COVID-19 hospitalization
Source: Commun Med (Lond). 2024 Feb 28;4:32. doi: 10.1038/s43856-024-00455-5 (PMC10901805; doi:10.1038/s43856-024-00455-5)
Supplement: Supplementary file 3 — Supplementary Data 1-7 [file 43856_2024_455_MOESM3_ESM.pdf]

Supplementary Data 1: Clinical characteristics of the study population, by deprivation status. Quintile 1 = most deprived, Quintile 5 = least deprived.

	All	SIMD					p-value
		Q1 Most Deprived	Q2	Q3	Q4	Q5 Least Deprived	
	N=252	N=101	N=56	N=31	N=23	N=41	
Demographics							
Age, years	55.0 ±12.0	55.1 ±11.2	54.1 ±12.1	57.4 ±13.8	52.8 ±13.5	55.5 ±11.4	0.6590
Sex							
Male	151 (60%)	68 (67%)	32 (57%)	13 (42%)	12 (52%)	26 (63%)	0.1148
Female	101 (40%)	33 (33%)	24 (43%)	18 (58%)	11 (48%)	15 (37%)	
Healthcare Worker	50 (20%)	21 (21%)	9 (16%)	5 (16%)	6 (26%)	9 (22%)	0.8205
Ethnicity							
White	227 (90%)	88 (87%)	54 (96%)	27 (87%)	21 (91%)	37 (90%)	0.6019
Asian	19 (8%)	10 (10%)	2 (4%)	3 (10%)	2 (9%)	2 (5%)	
Other	6 (2%)	3 (3%)	0 (0%)	1 (3%)	0 (0%)	2 (5%)	
Presenting Characteristics							
Weight, kg	90 ±19	92 ±21	94 ±20	88 ±16	88 ±19	85 ±16	0.2167
Height, cm	170 ±11	171 ±9	169 ±13	167 ±11	170 ±12	173 ±10	0.1426
**Body mass index, kg/m^2**	**31.3 ±6.9**	**31.3 ±6.3**	**33.0 ±7.4**	**31.8 ±7.1**	**30.8 ±8.3**	**28.7 ±5.9**	**0.0444**
Systolic blood pressure, mmHg	130 ±20	133 ±20	126 ±17	126 ±21	131 ±20	131 ±19	0.1767
Diastolic blood pressure, mmHg	78 ±13	79 ±13	76 ±14	72 ±13	80 ±15	79 ±11	0.0513
WHO Clinical severity score							
Hospitalized, no oxygen therapy	75 (30%)	30 (30%)	15 (27%)	6 (19%)	8 (35%)	16 (39%)	0.2781
Oxygen by mask or nasal prongs	119 (47%)	49 (49%)	23 (41%)	17 (55%)	12 (52%)	18 (44%)	
Non-invasive ventilation	34 (14%)	14 (14%)	12 (21%)	2 (6%)	3 (13%)	3 (7%)	
Mechanical ventilation	23 (9%)	7 (7%)	6 (11%)	6 (19%)	0 (0%)	4 (10%)	
COVID-19 diagnosis							
PCR test	252 (100%)	101 (100%)	56 (100%)	31 (100%)	23 (100%)	41 (100%)	1.0000
Nosocomial	12 (5%)	6 (6%)	3 (5%)	1 (3%)	1 (4%)	1 (2%)	0.9667
Radiology, chest radiograph or CT scan							

Supplementary Data 1: Clinical characteristics of the study population, by deprivation status. Quintile 1 = most deprived, Quintile 5 = least deprived.

	All	SIMD					p-value
		Q1 Most Deprived	Q2	Q3	Q4	Q5 Least Deprived	
Typical of COVID-19	174 (73%)	67 (71%)	41 (75%)	26 (87%)	13 (65%)	27 (71%)	0.8331
Atypical of COVID-19	20 (8%)	11 (12%)	3 (5%)	1 (3%)	2 (10%)	3 (8%)	
Unlikely	7 (3%)	3 (3%)	1 (2%)	1 (3%)	1 (5%)	1 (3%)	
Normal	37 (16%)	14 (15%)	10 (18%)	2 (7%)	4 (20%)	7 (18%)	
Acute COVID-19 therapy							
Oxygen	176 (70%)	70 (69%)	41 (73%)	25 (81%)	15 (65%)	25 (61%)	0.4380
Steroid	127 (50%)	53 (52%)	32 (57%)	17 (55%)	11 (48%)	14 (34%)	0.2120
Antiviral	63 (25%)	23 (23%)	18 (32%)	8 (26%)	5 (22%)	9 (22%)	0.7312
Non-invasive respiratory support	48 (19%)	19 (19%)	15 (27%)	5 (16%)	3 (13%)	6 (15%)	0.5524
Intensive care	32 (13%)	11 (11%)	10 (18%)	6 (19%)	1 (4%)	4 (10%)	0.3501
Invasive ventilation	22 (9%)	7 (7%)	6 (11%)	6 (19%)	0 (0%)	3 (7%)	0.1386
Intravenous inotrope	11 (4%)	3 (3%)	3 (5%)	2 (6%)	0 (0%)	3 (7%)	0.5711
Cardiovascular History							
Smoking							
Never	158 (63%)	62 (61%)	36 (64%)	17 (55%)	12 (52%)	31 (76%)	0.3480
Former	79 (31%)	30 (30%)	19 (34%)	12 (39%)	10 (43%)	8 (20%)	
Current	15 (6%)	9 (9%)	1 (2%)	2 (6%)	1 (4%)	2 (5%)	
Hypercholesterolaemia	118 (47%)	56 (55%)	23 (41%)	13 (42%)	10 (43%)	16 (39%)	0.2758
Hypertension	81 (32%)	37 (37%)	18 (32%)	9 (29%)	6 (26%)	11 (27%)	0.7704
**Diabetes mellitus**	**59 (23%)**	**31 (31%)**	**7 (12%)**	**9 (29%)**	**7 (30%)**	**5 (12%)**	**0.0239**
Chronic kidney disease	17 (7%)	10 (10%)	2 (4%)	2 (6%)	0 (0%)	3 (7%)	0.4487
CCS Angina Class							
No Angina	239 (95%)	94 (93%)	54 (96%)	31 (100%)	22 (96%)	38 (93%)	0.6092
Angina Class I-IV	13 (5%)	7 (7%)	2 (4%)	0 (0%)	1 (4%)	3 (7%)	
Heart failure	6 (2%)	2 (2%)	1 (2%)	0 (0%)	0 (0%)	3 (7%)	0.3360
Myocardial infarction	24 (10%)	14 (14%)	2 (4%)	3 (10%)	1 (4%)	4 (10%)	0.2804
Stroke or TIA	5 (2%)	2 (2%)	2 (4%)	1 (3%)	0 (0%)	0 (0%)	0.7698

Supplementary Data 1: Clinical characteristics of the study population, by deprivation status. Quintile 1 = most deprived, Quintile 5 = least deprived.

	All	SIMD					p-value
		Q1 Most Deprived	Q2	Q3	Q4	Q5 Least Deprived	
Peripheral vascular disease	3 (1%)	2 (2%)	0 (0%)	0 (0%)	0 (0%)	1 (2%)	0.8047
Previous PCI	11 (4%)	5 (5%)	1 (2%)	1 (3%)	0 (0%)	4 (10%)	0.3752
Previous CABG	4 (2%)	4 (4%)	0 (0%)	0 (0%)	0 (0%)	0 (0%)	0.3926
Cardiovascular disease and/or treatment	121 (48%)	55 (54%)	23 (41%)	16 (52%)	9 (39%)	18 (44%)	0.4225
Risk Scores							
ISARIC-4c in-hospital mortality risk, in %	12.7 ±10.9	13.6 ±12.0	12.5 ±9.5	14.5 ±12.4	10.3 ±11.0	10.3 ±8.5	0.3353
**Q-Risk 3, 10-year cardiovascular risk, in %**	**14.5 ±12.4**	**17.9 ±13.3**	**11.6 ±10.0**	**15.0 ±13.9**	**13.5 ±14.0**	**10.5 ±8.7**	**0.0124**
Charlson Comorbidity Index	2.0 ±2.0	2.3 ±2.3	1.7 ±1.6	2.0 ±2.0	1.7 ±1.4	1.6 ±1.7	0.2273
Pre-existing maintenance medication							
Aspirin	20 (8%)	9 (9%)	3 (5%)	2 (6%)	2 (9%)	4 (10%)	0.9197
Statin	67 (27%)	28 (28%)	15 (27%)	7 (23%)	6 (26%)	11 (27%)	0.9927
Beta-blocker	34 (13%)	22 (22%)	5 (9%)	3 (10%)	1 (4%)	3 (7%)	0.0556
**Angiotensin converting enzyme inhibitor**	**54 (21%)**	**33 (33%)**	**10 (18%)**	**4 (13%)**	**2 (9%)**	**5 (12%)**	**0.0121**
Oral anticoagulation	14 (6%)	3 (3%)	5 (9%)	2 (6%)	3 (13%)	1 (2%)	0.1711
Laboratory results, index admission							
Initial haemoglobin, g/l	141 ±16	143 ±16	140 ±18	138 ±18	142 ±11	140 ±14	0.4969
Initial platelet count, 10^9/l	236 ±87	230 ±80	242 ±85	229 ±87	225 ±75	255 ±108	0.5316
Initial white cell count, 10^9/l	7.43 ±4.83	7.35 ±3.57	7.39 ±3.04	7.52 ±3.52	6.12 ±1.94	8.38 ±9.41	0.5107
Peak D-Dimer, ng/ml	1723 ±5305	1846 ±6099	957 ±2409	1977 ±3362	294 ±306	2938 ±7936	0.6108
Minimum eGFR, ml/min/1.73m^2	80.2 ±25.7	80.6 ±25.9	80.9 ±23.9	76.6 ±27.0	92.0 ±18.3	74.7 ±28.7	0.1157
Peak hs-troponin I, ng/l	4.0 (2.0, 11.5)	4.0 (2.0, 12.5)	4.0 (2.0, 9.8)	4.0 (2.0, 16.0)	4.0 (2.0, 5.2)	4.0 (2.0, 13.0)	0.7746
Peak C-reactive protein, mg/l	116 (38, 189)	110 (32, 181)	143 (46, 196)	85 (57, 192)	67 (16, 150)	130 (21, 187)	0.4137
Peak HbA1c, mmol/mol	48.1 ±18.1	50.2 ±20.2	47.4 ±20.6	44.9 ±12.2	51.5 ±15.1	44.3 ±13.3	0.3617
**Initial albumin, g/l**	**34.1 ±5.0**	**34.4 ±5.1**	**33.4 ±4.7**	**32.3 ±5.9**	**36.0 ±3.6**	**35.0 ±5.1**	**0.0435**
Timelines							

Supplementary Data 1: Clinical characteristics of the study population, by deprivation status. Quintile 1 = most deprived, Quintile 5 = least deprived.

	All	SIMD					p-value
		Q1 Most Deprived	Q2	Q3	Q4	Q5 Least Deprived	
Hospitalized	225 (89%)	91 (90%)	51 (91%)	30 (97%)	18 (78%)	35 (85%)	0.2359
Duration of admission, days	6 (3, 12)	6 (3, 11)	5 (2, 12)	9 (4, 22)	5 (1, 8)	4 (3, 9)	0.0643
Symptom onset to primary outcome, days	66 (55, 77)	63 (52, 74)	68 (58, 77)	74 (63, 93)	65 (54, 72)	66 (54, 82)	0.1775
Diagnosis to primary outcome, days	62 (51, 74)	61 (50, 70)	64 (55, 71)	70 (57, 91)	58 (51, 64)	61 (50, 82)	0.2034

Deprivation status. Q1 = most deprived, Q5 = least deprived. Summaries are Mean ±SD, Median (IQR), or N (%). P-values from ANOVA, Kruskal-Wallis Test, or Fisher's Exact Test. Missing data: WHO Clinical severity score, n=1, Radiology, chest radiograph or CT scan, n=14, Q-Risk 3, 10-year cardiovascular risk, n=43, Peak D-Dimer, n=112, Minimum eGFR, n=1, Peak hs-troponin I, n=25, Peak ferritin, n=42, Peak C-reactive protein, n=1, Peak HbA1c, n=37.

Supplementary Data 2: Multisystem phenotyping by deprivation: serial electrocardiography, biomarkers of inflammation, metabolism, renal function, and haemostasis, and heart, lung, and kidney imaging at 28-60 days post-discharge.

	All	SIMD					p-value
		Q1<br>Most Deprived	Q2	Q3	Q4	Q5<br>Least Deprived	
Follow-up at 28-60 days post-discharge	186 (74%)	73 (72%)	36 (64%)	21 (68%)	21 (91%)	35 (85%)	p=0.0378
Electrocardiogram							
Myopericarditis criteria							
Admission	N=240	N=96	N=54	N=31	N=22	N=37	
	53 (22%)	26 (27%)	7 (13%)	8 (26%)	4 (18%)	8 (22%)	0.3376
Enrolment	N=228	N=94	N=47	N=28	N=20	N=39	
	68 (30%)	28 (30%)	13 (28%)	6 (21%)	7 (35%)	14 (36%)	0.7382
28-60 days post-discharge	N=167	N=66	N=34	N=19	N=14	N=34	
	40 (24%)	16 (24%)	9 (26%)	4 (21%)	3 (21%)	8 (24%)	0.9961
CT Chest 28-60 days post-discharge							
	N=168	N=64	N=31	N=21	N=18	N=34	
**Ground glass opacity and/or consolidation**	**70 (42%)**	**28 (44%)**	**18 (58%)**	**12 (57%)**	**3 (17%)**	**9 (26%)**	**0.0085**
Reticulation and/or architectural distortion	51 (30%)	22 (34%)	9 (29%)	8 (38%)	3 (17%)	9 (26%)	0.5833
Atelectasis	13 (8%)	5 (8%)	1 (3%)	1 (5%)	0 (0%)	6 (18%)	0.1820
Pulmonary arterial thrombus	5 (3%)	2 (3%)	0 (0%)	0 (0%)	0 (0%)	3 (9%)	0.3280
**Visual estimate of % of total lung area abnormal**	**14.1 ±19.1**	**14.6 ±18.8**	**20.0 ±21.9**	**17.9 ±21.8**	**4.7 ±11.0**	**10.4 ±17.1**	**0.0507**
CT coronary angiogram 28-60 days post-discharge							
	N=167	N=63	N=31	N=21	N=18	N=34	
Coronary calcium - Agatston score	140 ±486	277 ±762	65 ±129	116 ±174	24 ±65	37 ±98	0.0841
MESA percentile	60.5 ±34.3	71.1 ±29.7	50.1 ±41.5	62.4 ±35.5	43.1 ±28.3	46.3 ±32.6	0.0576
**Obstructive coronary artery disease**	**23 (14%)**	**13 (21%)**	**1 (4%)**	**6 (30%)**	**0 (0%)**	**3 (9%)**	**0.0128**

Supplementary Data 2: Multisystem phenotypying by deprivation: serial electrocardiography, biomarkers of inflammation, metabolism, renal function, and haemostasis, and heart, lung, and kidney imaging at 28-60 days post-discharge.

	All	SIMD					p-value
		Q1<br>Most Deprived	Q2	Q3	Q4	Q5<br>Least Deprived	
Follow-up at 28-60 days post-discharge	186 (74%)	73 (72%)	36 (64%)	21 (68%)	21 (91%)	35 (85%)	p=0.0378
FFR_{CT} patient-level (all coronary arteries) 28-60 days post-discharge							
	N=141	N=54	N=23	N=20	N=16	N=28	
Median FFR_{CT}	0.93 ±0.03	0.92 ±0.04	0.93 ±0.02	0.92 ±0.04	0.94 ±0.01	0.93 ±0.02	0.1595
**Minimum FFR_{CT}**	**0.80 ±0.10**	**0.78 ±0.10**	**0.81 ±0.08**	**0.76 ±0.14**	**0.83 ±0.10**	**0.83 ±0.06**	**0.0276**
**Minimum FFR_{CT} ≤0.8**	**53 (38%)**	**29 (54%)**	**8 (35%)**	**9 (45%)**	**4 (25%)**	**3 (11%)**	**0.0017**
Cardiac MRI 28–60 days post-discharge							
	N=151	N=61	N=28	N=18	N=17	N=27	
**LV end diastolic volume index, ml/m^2**	**76.0 ±17.3**	**75.7 ±17.3**	**72.5 ±14.3**	**69.0 ±15.6**	**72.9 ±13.8**	**86.6 ±19.1**	**0.0044**
**LV end systolic volume index, ml/m^2**	**35.4 ±13.0**	**35.8 ±13.5**	**33.0 ±12.6**	**31.8 ±9.9**	**31.4 ±7.6**	**41.6 ±14.8**	**0.0410**
LV ejection fraction, %	54.0 ±9.8	53.4 ±9.7	55.4 ±9.9	54.4 ±7.0	56.2 ±11.7	52.3 ±10.6	0.6704
LV mass, g	93.0 ±25.2	95.4 ±28.0	93.1 ±26.6	94.0 ±25.7	81.0 ±15.4	93.9 ±20.7	0.3747
**RV end diastolic volume index, ml/m^2**	**73.6 ±18.0**	**72.7 ±16.0**	**70.5 ±14.8**	**61.3 ±14.4**	**71.2 ±14.3**	**87.7 ±21.2**	**<0.0001**
**RV end systolic volume index, ml/m^2**	**36.2 ±11.5**	**35.2 ±10.7**	**35.3 ±10.6**	**30.3 ±8.5**	**36.2 ±8.9**	**42.7 ±14.3**	**0.0062**
RV ejection fraction, %	50.8 ±10.6	51.5 ±10.0	49.9 ±11.8	50.5 ±9.1	49.7 ±10.9	50.9 ±11.9	0.9607
Myocardial tissue characterisation							
	N=150	N=60	N=28	N=18	N=17	N=27	
Increased extracellular volume (>27.4%)	65 (48%)	28 (52%)	10 (42%)	11 (65%)	5 (36%)	11 (42%)	0.4501
	N=150	N=60	N=28	N=18	N=17	N=27	
Late gadolinium enhancement (scar)	27 (18%)	10 (17%)	3 (11%)	3 (17%)	3 (18%)	8 (30%)	0.4946
Myocardial inflammation (Lake Louise criteria)							

Supplementary Data 2: Multisystem phenotypying by deprivation: serial electrocardiography, biomarkers of inflammation, metabolism, renal function, and haemostasis, and heart, lung, and kidney imaging at 28-60 days post-discharge.

	All	SIMD					p-value
		Q1 Most Deprived	Q2	Q3	Q4	Q5 Least Deprived	
Follow-up at 28-60 days post-discharge	186 (74%)	73 (72%)	36 (64%)	21 (68%)	21 (91%)	35 (85%)	p=0.0378
No evidence (0/2)	17 (11%)	6 (10%)	7 (25%)	2 (11%)	0 (0%)	2 (7%)	0.2972
Probable (1/2)	71 (47%)	25 (42%)	11 (39%)	10 (56%)	11 (65%)	14 (52%)	
Definite (2/2)	62 (41%)	29 (48%)	10 (36%)	6 (33%)	6 (35%)	11 (41%)	
Renal MRI							
	N=134	N=55	N=26	N=16	N=16	N=21	
Average volume of right and left kidneys, ml	154 ±31	159 ±34	155 ±28	150 ±32	146 ±25	150 ±29	0.5917
**Average cortex T1 of right and left kidneys, ms**	**1545 ±63**	**1540 ±56**	**1551 ±65**	**1586 ±68**	**1521 ±69**	**1530 ±57**	**0.0421**
Average medulla T1 of right and left kidneys, ms	1933 ±70	1929 ±69	1947 ±78	1959 ±72	1912 ±55	1922 ±67	0.2868
Biomarkers at enrolment, central laboratory							
	N=244	N=97	N=53	N=30	N=23	N=41	
C-reactive protein, mg/l	4.7 (1.5, 18.2)	4.1 (1.7, 15.4)	5.4 (2.1, 22.6)	6.7 (2.3, 19.0)	6.8 (1.4, 21.4)	3.3 (0.9, 7.8)	0.5152
hs-troponin I, ng/l	3.2 (2.1, 5.5)	3.5 (2.1, 5.4)	3.2 (2.2, 5.3)	2.9 (1.9, 6.0)	2.9 (1.5, 3.5)	3.3 (2.2, 6.2)	0.7322
NT pro BNP, ng/l	106 (47, 243)	94 (45, 244)	122 (56, 349)	119 (65, 164)	72 (48, 262)	86 (34, 151)	0.4692
**Total bilirubin, µmol/l**	**5.5 (4.2, 7.9)**	**6.0 (4.5, 8.3)**	**5.3 (4.3, 6.6)**	**4.8 (3.3, 6.3)**	**6.4 (5.1, 7.9)**	**5.9 (4.2, 7.8)**	**0.0444**
Total Cholesterol, mmol/l	4.95 ±1.42	4.82 ±1.30	5.16 ±1.66	4.95 ±1.45	4.60 ±1.28	5.13 ±1.36	0.4951
Triglycerides, mmol/l	2.29 ±1.44	2.20 ±1.27	2.53 ±2.03	2.22 ±0.79	2.72 ±1.53	2.02 ±1.11	0.3256
Biomarkers at 28-60 days post-discharge, central laboratory (control group samples from enrolment visit)							
	N=180	N=70	N=35	N=21	N=19	N=35	
C-reactive protein, mg/l	2.1 (1.2, 4.1)	2.0 (0.9, 4.0)	2.3 (1.5, 4.4)	2.2 (1.3, 3.2)	2.1 (0.9, 5.2)	1.7 (1.0, 4.9)	0.7211
hs-troponin I, ng/l	2.1 (1.3, 3.9)	2.1 (1.3, 3.8)	2.1 (1.4, 3.5)	1.8 (1.2, 3.1)	1.5 (0.9, 3.2)	2.9 (1.8, 5.5)	0.2394
NT pro BNP, ng/l	80 (52, 183)	79 (51, 249)	127 (49, 243)	78 (57, 110)	73 (44, 134)	82 (55, 131)	0.5870

Multisystem phenotypying by deprivation status Q1 = most deprived, Q5 = least deprived. Summaries are Mean ±SD, Median (IQR), or N (%). P-values from ANOVA, Kruskal-Wallis Test, or Fisher's Exact Test. Missing data: Electrocardiogram: admission – n=12, enrolment – n=24, 28-60 days – n=1. CT coronary angiogram: Agatston score – n=4; CAD-RADS score – n=5; FFRCT – n=26; Cardiovascular magnetic resonance imaging 28-60 days post-discharge: left ventricular end-diastolic volume index, left ventricular end-systolic volume index, left ventricular ejection fraction, left ventricular strain, left ventricular mass – n=2; right ventricular end-diastolic volume index, right ventricular systolic volume index, n=4; right ventricular ejection fraction, n=3; global extracellular volume – n=15; late gadolinium enhancement distribution – n=12. Missing data in blood biomarkers (enrolment and 28-60 days)– eGFR – n=10, n=9; C-reactive protein – n=42, n=5; High sensitivity troponin I – n=45, n=8; NT proBNP – n=45, n=11; Total bilirubin (enrolment) – n=13, Total cholesterol, triglycerides, HDL cholesterol (enrolment) – n=42.

Supplementary Data 3: Clinical characteristics of the study population, by attendance at Visit 2.

	All	SIMD		p-value
		V2 Not Attended	V2 Attended	
	N=252	N=66	N=186	
Demographics				
Age, years	55.0 ±12.0	56.6 ±13.2	54.4 ±11.5	p=0.2017
Sex				
Male	151 (60%)	46 (70%)	105 (56%)	p=0.0789
Female	101 (40%)	20 (30%)	81 (44%)	
SIMD Quintile				
Q1 -	101 (40%)	28 (42%)	73 (39%)	
Q2	56 (22%)	20 (30%)	36 (19%)	
Q3	31 (12%)	10 (15%)	21 (11%)	p=0.0378
Q4	23 (9%)	2 (3%)	21 (11%)	
Q5	41 (16%)	6 (9%)	35 (19%)	
Healthcare Worker	50 (20%)	8 (12%)	42 (23%)	p=0.0740
Ethnicity				
White	227 (90%)	61 (92%)	166 (89%)	
Asian	19 (8%)	5 (8%)	14 (8%)	p=0.4572
Other	6 (2%)	0 (0%)	6 (3%)	
Presenting Characteristics				
Weight, kg	90 ±19	93 ±21	90 ±18	p=0.2895
Height, cm	170 ±11	172 ±10	170 ±11	p=0.1208
Body mass index, kg/m^2	31.3 ±6.9	31.2 ±6.5	31.3 ±7.0	p=0.9376
Body surface area, m^2	2.1 ±0.2	2.1 ±0.3	2.0 ±0.2	p=0.1919
Heart Rate, bpm	95 ±19	94 ±19	96 ±19	p=0.4182
Systolic blood pressure, mmHg	130 ±20	132 ±17	130 ±20	p=0.3626
Diastolic blood pressure, mmHg	78 ±13	78 ±14	77 ±13	p=0.7435
Peripheral oxygen saturation, %	94 ±6	94 ±5	93 ±6	p=0.3343
Respiratory rate, /min	23 ±8	23 ±6	23 ±9	p=0.9265
WHO Clinical severity score				

Supplementary Data 3: Clinical characteristics of the study population, by attendance at Visit 2.

	All	SIMD		p-value
		V2 Not Attended	V2 Attended	
Hospitalized, no oxygen therapy	75 (30%)	16 (24%)	59 (32%)	p=0.3093
Oxygen by mask or nasal prongs	119 (47%)	34 (52%)	85 (46%)	
Non-invasive ventilation	34 (14%)	12 (18%)	22 (12%)	
Mechanical ventilation	23 (9%)	4 (6%)	19 (10%)	
COVID-19 diagnosis				
PCR test	252 (100%)	66 (100%)	186 (100%)	p=1.0000
Nosocomial	12 (5%)	4 (6%)	8 (4%)	p=0.5187
Radiology, chest radiograph or CT scan				
Typical of COVID-19	174 (73%)	47 (71%)	127 (74%)	p=0.5690
Atypical of COVID-19	20 (8%)	4 (6%)	16 (9%)	
Unlikely	7 (3%)	3 (5%)	4 (2%)	
Normal	37 (16%)	12 (18%)	25 (15%)	
Acute COVID-19 therapy				
Oxygen	176 (70%)	50 (76%)	126 (68%)	p=0.2749
Steroid	127 (50%)	38 (58%)	89 (48%)	p=0.1983
Antiviral	63 (25%)	19 (29%)	44 (24%)	p=0.4123
Non-invasive respiratory support	48 (19%)	13 (20%)	35 (19%)	p=0.8570
Intensive care	32 (13%)	4 (6%)	28 (15%)	p=0.0832
Invasive ventilation	22 (9%)	4 (6%)	18 (10%)	p=0.4549
Intravenous inotrope	11 (4%)	0 (0%)	11 (6%)	p=0.0717
Cardiovascular History				
Smoking				
Never	158 (63%)	37 (56%)	121 (65%)	p=0.3717
Former	79 (31%)	25 (38%)	54 (29%)	
Current	15 (6%)	4 (6%)	11 (6%)	
Hypercholesterolaemia	118 (47%)	30 (45%)	88 (47%)	p=0.8860
Hypertension	81 (32%)	20 (30%)	61 (33%)	p=0.7606
Diabetes mellitus	59 (23%)	22 (33%)	37 (20%)	p=0.0413
Chronic kidney disease	17 (7%)	9 (14%)	8 (4%)	p=0.0184

Supplementary Data 3: Clinical characteristics of the study population, by attendance at Visit 2.

	All	SIMD		p-value
		V2 Not Attended	V2 Attended	
CCS Angina Class				
No Angina	239 (95%)	58 (88%)	181 (97%)	p=0.0063
Angina Class I-IV	13 (5%)	8 (12%)	5 (3%)	
Heart failure	6 (2%)	1 (2%)	5 (3%)	p=1.0000
Myocardial infarction	24 (10%)	7 (11%)	17 (9%)	p=0.8075
Stroke or TIA	5 (2%)	1 (2%)	4 (2%)	p=1.0000
Peripheral vascular disease	3 (1%)	2 (3%)	1 (1%)	p=0.1687
Previous PCI	11 (4%)	1 (2%)	10 (5%)	p=0.2973
Previous CABG	4 (2%)	2 (3%)	2 (1%)	p=0.2812
Cardiovascular disease and/or treatment	121 (48%)	35 (53%)	86 (46%)	p=0.3904
Risk Scores				
ISARIC-4c in-hospital mortality risk, in %	12.7 ±10.9	15.8 ±12.0	11.6 ±10.4	p=0.0071
Q-Risk 3, 10-year cardiovascular risk, in %	14.5 ±12.4	18.3 ±15.5	13.0 ±10.7	p=0.0056
Charlson Comorbidity Index	2.0 ±2.0	2.6 ±2.5	1.7 ±1.7	p=0.0033
Pre-existing maintenance medication				
Aspirin	20 (8%)	8 (12%)	12 (6%)	p=0.1831
Statin	67 (27%)	19 (29%)	48 (26%)	p=0.6303
Beta-blocker	34 (13%)	10 (15%)	24 (13%)	p=0.6765
Angiotensin converting enzyme inhibitor	54 (21%)	16 (24%)	38 (20%)	p=0.6006
Angiotensin receptor blocker	18 (7%)	6 (9%)	12 (6%)	p=0.5777
Oral anticoagulation	14 (6%)	5 (8%)	9 (5%)	p=0.5310
Laboratory results, index admission				
Initial hemoglobin, g/l	141 ±16	141 ±18	141 ±16	p=0.9145
Initial platelet count, 10^9/l	236 ±87	235 ±86	237 ±87	p=0.8624
Initial white cell count, 10^9/l	7.43 ±4.83	7.67 ±3.64	7.35 ±5.20	p=0.6471
Initial lymphocyte count, 10^9/l	1.42 ±3.73	1.17 ±0.66	1.51 ±4.32	p=0.5285
Peak D-Dimer, ng/ml	1723 ±5305	1115 ±3508	1896 ±5716	p=0.4714

Supplementary Data 3: Clinical characteristics of the study population, by attendance at Visit 2.

	All	SIMD		p-value
		V2 Not Attended	V2 Attended	
Minimum eGFR, ml/min/1.73m^2	80.2 ±25.7	77.3 ±23.9	81.3 ±26.3	p=0.2741
Acute kidney injury	19 (14%)	-	19 (14%)	p=1.0000
Peak hs-troponin I, ng/l	4.0 (2.0, 11.5)	4.0 (2.0, 12.0)	4.0 (2.0, 11.0)	p=0.7937
Peak ferritin, µg/l	360 (148, 919)	518 (121, 904)	355 (151, 932)	p=0.9730
Peak C-reactive protein, mg/l	116 (38, 189)	137 (48, 215)	110 (36, 183)	p=0.2419
Peak HbA1c, mmol/mol	48.1 ±18.1	50.6 ±18.9	47.2 ±17.7	p=0.2287
Initial albumin, g/l	34.1 ±5.0	33.6 ±4.3	34.3 ±5.3	p=0.3369
Timelines				
Hospitalized	225 (89%)	61 (92%)	164 (88%)	p=0.4871
Duration of admission, days	6 (3, 12)	8 (3, 14)	5 (2, 10)	p=0.0394

Supplementary Data 4: Health status, illness perception, anxiety and depression, and physical function by deprivation status.

	All	SIMD					p-value
		Q1<br>Most Deprived	Q2	Q3	Q4	Q5<br>Least Deprived	
Enrolment	N=242	N=97	N=53	N=30	N=22	N=40	
28-60 days post-discharge	N=184	N=73	N=35	N=21	N=20	N=35	
Health-related Quality of Life, EQ-5D-5L							
Heath Utility Score at enrolment	0.71 ±0.24	0.72 ±0.25	0.67 ±0.24	0.69 ±0.28	0.76 ±0.19	0.76 ±0.23	0.3312
Heath Utility Score at 28-60 days post-discharge	**0.78 ±0.22**	**0.76 ±0.20**	**0.72 ±0.21**	**0.70 ±0.30**	**0.85 ±0.18**	**0.87 ±0.17**	**0.0084**
Your Health Today VAS at enrolment	61.18 ±21.87	60.19 ±23.30	62.00 ±18.80	59.93 ±21.16	57.05 ±21.14	65.72 ±23.19	0.5800
Your Health Today VAS at 28-60 days post-discharge	73.58 ±18.26	72.89 ±18.43	70.71 ±18.56	69.90 ±19.36	76.10 ±16.53	78.63 ±17.65	0.3026
Brief Illness Perception Questionnaire Score							
At enrolment	41.7 ±13.2	41.4 ±13.9	43.2 ±11.9	44.8 ±13.3	43.9 ±11.3	37.0 ±13.3	0.0856
**At 28-60 days post-discharge**	**36.6 ±14.5**	**38.6 ±14.2**	**38.0 ±12.8**	**43.6 ±13.2**	**34.3 ±16.2**	**28.0 ±13.1**	**0.0004**
Anxiety and Depression, PHQ-4							
Anxiety score at enrolment	2.23 ±2.13	2.02 ±2.02	2.63 ±2.12	2.93 ±2.23	2.14 ±2.19	1.74 ±2.20	0.0847
**Anxiety score at 28-60 days post-discharge**	**1.84 ±2.01**	**1.79 ±1.96**	**2.50 ±2.08**	**2.52 ±2.16**	**1.42 ±2.27**	**1.09 ±1.52**	**0.0184**
**Depression score at enrolment**	**2.28 ±1.96**	**2.15 ±1.95**	**2.37 ±1.91**	**3.13 ±1.94**	**2.55 ±1.95**	**1.69 ±1.94**	**0.0380**
**Depression score at 28-60 days post-discharge**	**1.75 ±1.90**	**1.87 ±1.94**	**2.38 ±1.95**	**2.38 ±1.99**	**1.05 ±1.78**	**0.88 ±1.32**	**0.0024**
**Total score at enrolment**	**4.52 ±3.85**	**4.18 ±3.71**	**5.00 ±3.82**	**6.07 ±3.84**	**4.68 ±3.86**	**3.44 ±3.99**	**0.0474**
**Total score at 28-60 days post-discharge**	**3.59 ±3.70**	**3.66 ±3.71**	**4.88 ±3.83**	**4.90 ±3.99**	**2.47 ±3.72**	**1.97 ±2.58**	**0.0038**
Physical Function							
IPAQ score at enrolment							
Low	**163 (74%)**	**72 (85%)**	**39 (80%)**	**16 (57%)**	**16 (80%)**	**20 (53%)**	
Moderate	**28 (13%)**	**5 (6%)**	**6 (12%)**	**4 (14%)**	**1 (5%)**	**12 (32%)**	**0.0012**
High	**29 (13%)**	**8 (9%)**	**4 (8%)**	**8 (29%)**	**3 (15%)**	**6 (16%)**	
IPAQ score at 28-60 days post-discharge							
Low	74 (48%)	33 (55%)	16 (53%)	9 (53%)	9 (50%)	7 (23%)	
Moderate	48 (31%)	17 (28%)	6 (20%)	6 (35%)	7 (39%)	12 (40%)	0.0974
High	33 (21%)	10 (17%)	8 (27%)	2 (12%)	2 (11%)	11 (37%)	
DASI score at enrolment	19.4 ±18.0	20.7 ±20.1	15.9 ±14.4	15.3 ±12.1	20.0 ±18.5	23.8 ±19.7	0.1646

Supplementary Data 4: Health status, illness perception, anxiety and depression, and physical function by deprivation status.

	All	SIMD					p-value
		Q1 Most Deprived	Q2	Q3	Q4	Q5 Least Deprived	
**DASI score at 28-60 days post-discharge**	**25.3 ±18.0**	**22.2 ±17.5**	**21.4 ±16.5**	**21.9 ±14.3**	**29.7 ±18.8**	**35.2 ±18.6**	**0.0022**
DASI VO_2 max estimate at enrolment	18.0 ±7.7	18.5 ±8.6	16.4 ±6.2	16.2 ±5.2	18.2 ±8.0	19.8 ±8.5	0.1646
**DASI VO_2 max estimate at 28-60 days post-discharge**	**20.5 ±7.7**	**19.2 ±7.5**	**18.8 ±7.1**	**19.0 ±6.2**	**22.4 ±8.1**	**24.7 ±8.0**	**0.0022**

Health status, illness perception, anxiety and depression, and physical function by deprivation status. Q1 = most deprived, Q5 = least deprived. Summaries are Mean ±SD, Median (IQR), or N (%). P-values from ANOVA, Kruskal-Wallis Test, or Fisher's Exact Test.

Supplementary Data 5: Linear mixed effects regression models for patient reported outcomes in relation to SIMD.

	Estimate (95% CI)	p-value	Interaction p-value
EQ-5D VAS			
Trend per SIMD Quintile at enrolment	.9 (-0.8, 2.6)	0.2911	0.5785
Trend per SIMD Quintile at 28-60 days post-discharge	1.5 (-0.4, 3.3)	0.1213	
Change from enrolment to 28-60 days (SIMD Q1)	11.5 (7.4, 15.5)	**<0.0001**	
Change from enrolment to 28-60 days (SIMD Q5)	13.6 (8.0, 19.2)	**<0.0001**	
EQ-5D Health Utility			
Trend per SIMD Quintile at enrolment	0.014 (-0.005, 0.034)	0.1422	0.2088
Trend per SIMD Quintile at 28-60 days post-discharge	0.028 (0.007, 0.049)	**0.0093**	
Change from enrolment to 28-60 days (SIMD Q1)	0.037 (-0.007, 0.081)	0.1019	
Change from enrolment to 28-60 days (SIMD Q5)	0.090 (0.029, 0.150)	**0.0037**	
Brief Illness Perception Questionnaire Score			
Trend per SIMD Quintile at enrolment	-0.73 (-1.86, 0.41)	0.2094	**0.0186**
Trend per SIMD Quintile at 28-60 days post-discharge	-2.19 (-3.41, -0.97)	0.0004	
Change from enrolment to 28-60 days (SIMD Q1)	-2.94 (-5.55, -0.33)	0.0274	
Change from enrolment to 28-60 days (SIMD Q5)	-8.79 (-12.38, -5.21)	**<0.0001**	
PHQ4 Total Score			
Trend per SIMD Quintile at enrolment	-0.11 (-0.43, 0.21)	0.5041	**0.0408**
Trend per SIMD Quintile at 28-60 days post-discharge	-0.46 (-0.81, -0.12)	0.0084	
Change from enrolment to 28-60 days (SIMD Q1)	-0.27 (-1.00, 0.45)	0.4642	
Change from enrolment to 28-60 days (SIMD Q5)	-1.69 (-2.69, -0.69)	**0.0009**	
PHQ4 Anxiety Score			
Trend per SIMD Quintile at enrolment	-0.05 (-0.23, 0.12)	0.5533	0.1683
Trend per SIMD Quintile at 28-60 days post-discharge	-0.19 (-0.38, 0.00)	0.0550	
Change from enrolment to 28-60 days (SIMD Q1)	-0.14 (-0.54, 0.27)	0.5062	
Change from enrolment to 28-60 days (SIMD Q5)	-0.67 (-1.23, -0.11)	0.0190	
PHQ4 Depression Score			

Trend per SIMD Quintile at enrolment	-0.06 (-0.22, 0.11)	0.5081	**0.0203**
Trend per SIMD Quintile at 28-60 days post-discharge	-0.28 (-0.45, -0.10)	**0.0023**	
Change from enrolment to 28-60 days (SIMD Q1)	-0.15 (-0.54, 0.25)	0.4695	
Change from enrolment to 28-60 days (SIMD Q5)	-1.03 (-1.58, -0.48)	**0.0002**	
IPAQ High Physical Activity (trends and changes reported as odds ratios)			
Trend per SIMD Quintile at enrolment	1.37 (0.54, 3.48)	0.5030	0.7714
Trend per SIMD Quintile at 28-60 days post-discharge	1.22 (0.62, 2.41)	0.5702	
Change from enrolment to 28-60 days (SIMD Q1)	12.01 (1.24, 116.53)	**0.0320**	
Change from enrolment to 28-60 days (SIMD Q5)	7.42 (0.82, 66.67)	0.0738	
IPAQ Moderate/High Physical Activity (trends and changes reported as odds ratios)			
Trend per SIMD Quintile at enrolment	1.58 (1.24, 2.00)	**0.0002**	0.5644
Trend per SIMD Quintile at 28-60 days post-discharge	1.45 (1.16, 1.81)	**0.0011**	
Change from enrolment to 28-60 days (SIMD Q1)	2.10 (1.09, 4.04)	**0.0265**	
Change from enrolment to 28-60 days (SIMD Q5)	1.50 (0.66, 3.39)	0.3345	
DASI Score			
Trend per SIMD Quintile at enrolment	0.84 (-0.59, 2.26)	0.2503	**0.0027**
Trend per SIMD Quintile at 28-60 days post-discharge	3.23 (1.70, 4.77)	**<0.0001**	
Change from enrolment to 28-60 days (SIMD Q1)	2.00 (-1.34, 5.34)	0.2410	
Change from enrolment to 28-60 days (SIMD Q5)	11.59 (7.01, 16.18)	**<0.0001**	

Models include random effects for subjects, fixed effects for age and sex, fixed effects for study visits, fixed effects for the trend in outcome per quintile increase in SIMD (where Q1 = most deprived, Q5 = least deprived), and an interaction between SIMD trend and visit. Model-derived estimates reported for trend across SIMD quintiles at each study visit, and for mean changes between study visits in most and least deprived SIMD quintiles. P-values for SIMD-by-visit interactions also reported.

Supplementary Data 6: Linear mixed effects regression model data for patient reported outcomes in relation to SIMD.

**Outcome**	**SIMD Quintile**	**Visit**	Predicted	Lower Control Limit	Upper Control Limit
Brief Illness Perception Questionnaire Score	1	1	38.99570	31.38063	46.61077
Brief Illness Perception Questionnaire Score	2	1	38.26895	30.78938	45.74853
Brief Illness Perception Questionnaire Score	3	1	37.54221	30.02728	45.05714
Brief Illness Perception Questionnaire Score	4	1	36.81546	29.09669	44.53424
Brief Illness Perception Questionnaire Score	5	1	36.08872	28.01034	44.16710
Brief Illness Perception Questionnaire Score	1	2	36.05638	28.37306	43.73970
Brief Illness Perception Questionnaire Score	2	2	33.86594	26.34798	41.38390
Brief Illness Perception Questionnaire Score	3	2	31.67550	24.12602	39.22498
Brief Illness Perception Questionnaire Score	4	2	29.48506	21.70957	37.26055
Brief Illness Perception Questionnaire Score	5	2	27.29462	19.11474	35.47449
Patient health questionnaire-4 (Total)	1	1	4.551530	2.4128961	6.690163
Patient health questionnaire-4 (Total)	2	1	4.442801	2.3422210	6.543381
Patient health questionnaire-4 (Total)	3	1	4.334072	2.2234812	6.444664
Patient health questionnaire-4 (Total)	4	1	4.225344	2.0573426	6.393345
Patient health questionnaire-4 (Total)	5	1	4.116615	1.8473997	6.385831
Patient health questionnaire-4 (Total)	1	2	4.280297	2.1171088	6.443486
Patient health questionnaire-4 (Total)	2	2	3.816519	1.7030642	5.929974
Patient health questionnaire-4 (Total)	3	2	3.352741	1.2334070	5.472074
Patient health questionnaire-4 (Total)	4	2	2.888962	0.7085872	5.069337
Patient health questionnaire-4 (Total)	5	2	2.425184	0.1330074	4.717360
Patient health questionnaire-4 (Depression)	1	1	2.2294104	1.1532222	3.305599
Patient health questionnaire-4 (Depression)	2	1	2.1742923	1.1181193	3.230465
Patient health questionnaire-4 (Depression)	3	1	2.1191742	1.0579905	3.180358
Patient health questionnaire-4 (Depression)	4	1	2.0640560	0.9731803	3.154932
Patient health questionnaire-4 (Depression)	5	1	2.0089379	0.8656103	3.152266
Patient health questionnaire-4 (Depression)	1	2	2.0828907	0.9926975	3.173084
Patient health questionnaire-4 (Depression)	2	2	1.8066857	0.7431845	2.870187
Patient health questionnaire-4 (Depression)	3	2	1.5304806	0.4643375	2.596624
Patient health questionnaire-4 (Depression)	4	2	1.2542756	0.1563681	2.352183
Patient health questionnaire-4 (Depression)	5	2	0.9780706	-0.1783262	2.134467
Duke Activity Status Index Score	1	1	42.06898	32.58852	51.54943
Duke Activity Status Index Score	2	1	42.90454	33.59759	52.21150
Duke Activity Status Index Score	3	1	43.74011	34.39032	53.08989
Duke Activity Status Index Score	4	1	44.57567	34.96962	54.18173
Duke Activity Status Index Score	5	1	45.41124	35.35177	55.47071
Duke Activity Status Index Score	1	2	44.06840	34.50237	53.63443
Duke Activity Status Index Score	2	2	47.30254	37.94549	56.65960
Duke Activity Status Index Score	3	2	50.53669	41.13995	59.93343

Supplementary Data 6: Linear mixed effects regression model data for patient reported outcomes in relation to SIMD.

Duke Activity Status Index Score	4	2	53.77083	44.08880	63.45287
Duke Activity Status Index Score	5	2	57.00498	46.81265	67.19730

Supplementary Data 7: Clinical outcomes by deprivation status.

	All	SIMD					p-value
		Q1 Most Deprived	Q2	Q3	Q4	Q5 Least Deprived	
	N=252	N=101	N=56	N=31	N=23	N=41	
Duration of Follow-up							
Days to Visit 3 or death	428 (385, 538)	426 (369, 498)	430 (408, 545)	429 (386, 545)	420 (382, 437)	436 (392, 566)	0.3504
Outcomes							
Death or Hospitalization (Any Cause)	56 (22%)	29 (29%)	11 (20%)	4 (13%)	2 (9%)	10 (24%)	0.1812
Death (Any Cause)	4 (2%)	2 (2%)	0 (0%)	1 (3%)	1 (4%)	0 (0%)	0.5071
Hospitalization (Any Cause)	54 (21%)	28 (28%)	11 (20%)	4 (13%)	1 (4%)	10 (24%)	0.1235
Cardiovascular Outcomes							
Myocardial infarction	1 (0%)	0 (0%)	1 (2%)	0 (0%)	0 (0%)	0 (0%)	0.4861
Cerebrovascular accident	4 (2%)	2 (2%)	1 (2%)	0 (0%)	0 (0%)	1 (2%)	0.9015
Heart Failure	3 (1%)	2 (2%)	1 (2%)	0 (0%)	0 (0%)	0 (0%)	0.7650
New atrial fibrillation	4 (2%)	1 (1%)	0 (0%)	1 (3%)	0 (0%)	2 (5%)	0.3031
Respiratory Outcomes							
Pulmonary fibrosis	14 (6%)	6 (6%)	3 (5%)	3 (10%)	0 (0%)	2 (5%)	0.6625
Pulmonary embolism	5 (2%)	2 (2%)	0 (0%)	0 (0%)	0 (0%)	3 (7%)	0.0888
Long-term oxygen therapy	2 (1%)	0 (0%)	0 (0%)	1 (3%)	0 (0%)	1 (2%)	0.2739
Secondary Care (Outpatients)							
Any Outpatient Referral	170 (67%)	69 (68%)	37 (66%)	24 (77%)	12 (52%)	28 (68%)	0.1267
Acute COVID-19 (<28 days)	20 (8%)	8 (8%)	3 (5%)	5 (16%)	0 (0%)	4 (10%)	0.2306
**Ongoing COVID-19 (28-84 days)**	**35 (14%)**	**13 (13%)**	**3 (5%)**	**9 (29%)**	**3 (13%)**	**7 (17%)**	**0.0438**
Long COVID-19 (>84 days)	100 (40%)	42 (42%)	30 (54%)	10 (32%)	5 (22%)	13 (32%)	0.0920

Clinical outcomes by deprivation status. Q1 = most deprived, Q5 = least deprived. Duration of follow-up summarised as Median (IQR), and compared between groups using Kruskal-Wallis tests. Clinical outcomes summarised as number and percentage with at least one event, and compared between groups using log rank test of time to first event.
